# Optimum level of NEDD4 and its interaction with nsP3 are crucial to facilitate efficient Chikungunya virus (CHIKV) infection

**DOI:** 10.1099/jgv.0.002136

**Published:** 2025-08-11

**Authors:** Suchanda Verma, Sanchari Chatterjee, Supriya Suman Keshry, Ajit Kumar Dhal, Bijita Bhowmick, Janu Newar, Soma Chattopadhyay, Archana Ghatak

**Affiliations:** 1 School of Biotechnology, Kalinga Institute of Industrial Technology (KIIT) University, Bhubaneswar, India; 2 Institute of Life Sciences, Bhubaneswar, India; 3 Regional Centre for Biotechnology, Faridabad, India; 4 Center for Cancer Research, National Cancer Institute at Frederick, Frederick, MD, 21702, USA; 5 Department of Pediatrics, University of California, San Diego, USA

**Keywords:** Chikungunya, host protein, infection, interaction, NEDD4

## Abstract

Chikungunya is a febrile infection caused by the Chikungunya virus (CHIKV), an alphavirus which has emerged as a serious public health problem globally. Despite extensive research, our understanding of different host factors facilitating effective CHIKV infection is not clear yet. NEDD4, a member of the E3 ubiquitin ligase, is one such protein. Here, the importance of NEDD4 has been explored during CHIKV infection *in vitro*. It was observed that the level of NEDD4 is downregulated after CHIKV infection. Interestingly, the CHIKV-nsP3 level and the viral load were decreased significantly when NEDD4 was silenced, while a 93% decrease in the viral load was observed in the case of NEDD4 overexpression, indicating the importance of an optimum level of NEDD4 for effective CHIKV infection. Further study revealed that there was interaction between the NEDD4 and CHIKV-nsP3 proteins through co-immunoprecipitation (CO-IP) during CHIKV infection. Additionally, *in silico* data illustrated that the WW domain of NEDD4 can bind to the nsP3, as well as the macrodomain of nsP3 (nsp3-MD) of CHIKV. These data were further confirmed by the pull-down assay with purified nsP3-MD. The finding suggested that the host protein NEDD4 might interact directly with nsP3-MD during the CHIKV infection. However, the presence of a faint band of NEDD4 along with nsP3-MD in the pull-down assay may indicate the involvement of some other residues for this interaction. These *in silico* data were further confirmed by the CO-IP experiments, where all domains of nsP3, MD (macrodomain), AUD (alphavirus unique domain) and HVD (hypervariable domain) were found to interact with NEDD4. Additional experiments with a truncated form of MD, MD1 (1–100 residues of amino acid), revealed that this region is not able to maintain the interaction with NEDD4, indicating the crucial role of the C-terminal region of MD for this binding. In conclusion, these findings offer valuable insights about the importance of NEDD4 during CHIKV infection and the residues of nsP3 for its interaction, which might be useful to design future therapeutics against CHIKV.

## Introduction

Chikungunya virus (CHIKV) is a member of the *Alphavirus* genus of *Togaviridae* family [1]. Its single-stranded genomic RNA (11.8 kb) encodes four non-structural (nsP1–4) and five structural proteins [2]. Among these, the function of nsP3 is least understood. It serves as an accessory protein in the synthesis of minus-strand and sub-genomic RNA [3]. In addition, it plays a significant role in suppressing the host’s antiviral response [4]. Even so, the precise molecular mechanism and the degree of involvement of nsP3 in viral infection are still not clear. This protein is composed of three domains: the N-terminal macrodomain (MD, 1–159 aa), the alphavirus unique domain (AUD, 160–326 aa) and the C-terminal unstructured hypervariable domain (HVD, 327–529 aa). The macrodomain of nsP3 (nsP3-MD) or X domain is highly conserved across species [5]. nsP3-MD demonstrated ADP-ribose 1″-phosphate phosphatase and ADP-ribosyl hydrolase activities [6]. These activities are thought to be important for controlling host antiviral responses [7]. The AUD domain plays a crucial role in the viral life cycle, particularly in RNA synthesis and virus assembly [8]. The nsP3 C-terminal domain also contributes towards regulation of the host defence mechanism through its direct interaction with the host proteins, like G3BP, FHL1 and NAP1L1/L4 [9,13]. Unlike the C-terminal domain, the nsP3-MD has not yet been reported to interact with host proteins.

The ubiquitin system is a post-translational protein modification system, which plays a crucial role in regulating protein degradation, signalling pathways, immune responses and cellular functions [14]. E3 ubiquitin ligases are important components of this system, as these ligases directly interact with the protein substrates that are modified by ubiquitination [15]. NEDD4 is a member of the E3 ubiquitin-protein ligase, known to ubiquitinate viral matrix proteins for a number of viruses [16,17] and interacts with components of the endocytic machinery required for viral budding [18] and release for a number of viruses [19].

NEDD4 was shown to interact with CHIKV proteins nsP1, nsP3 and nsP4 through co-transfection of HEK293T cells with plasmids expressing HA-tagged ESCRT proteins and FLAG-tagged CHIKV proteins [20]. However, its role in CHIKV infection is yet to be studied. Hence, in this investigation, the importance of the NEDD4 protein in CHIKV infection was explored.

## Results

### CHIKV infection leads to downregulation of NEDD4 expression in host cells

**Fig. 1.**
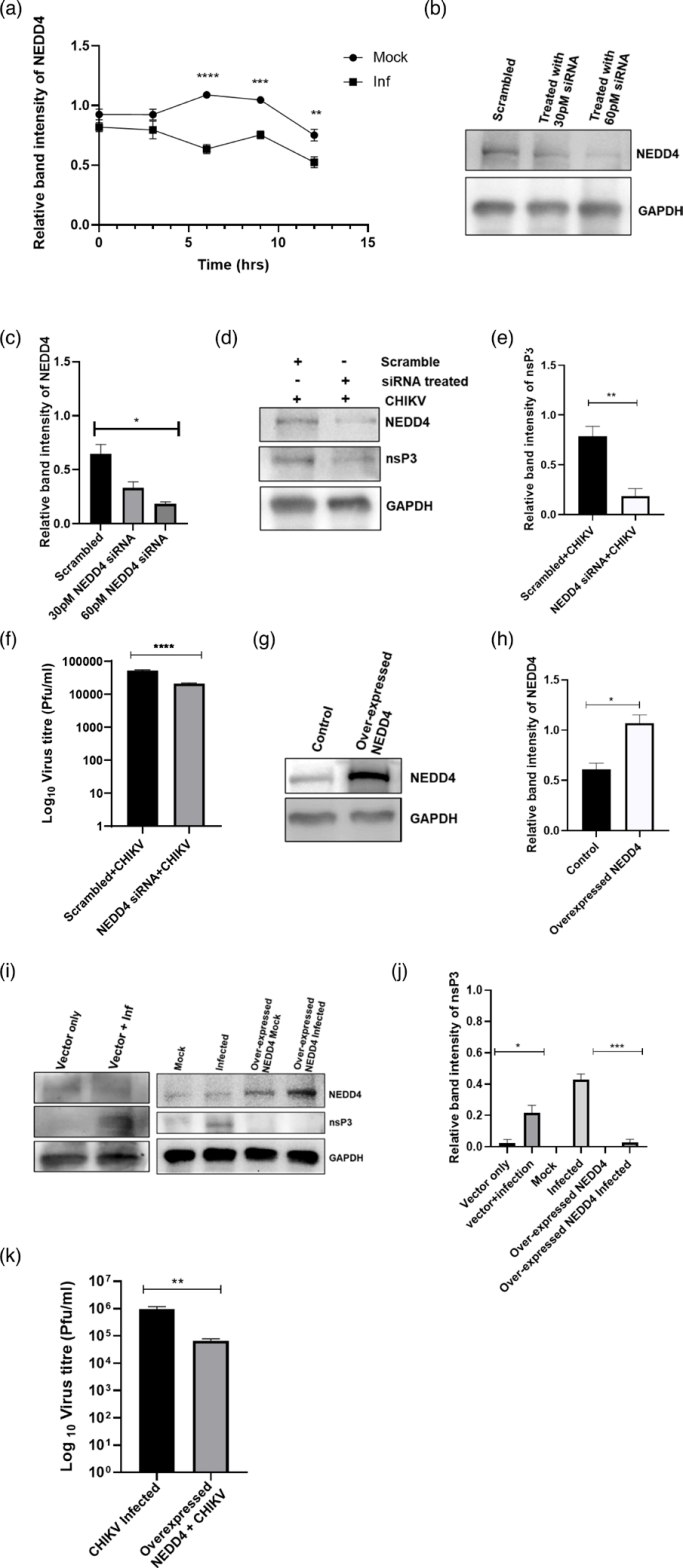
Optimum level of NEDD4 is crucial for CHIKV infection. Vero cells were infected with CHIKV, and NEDD4 expression was checked at different time points. (**a**) Line diagram depicting the relative band intensity of NEDD4 in mock and infected cells at different time points. (**b**) The HEK293T cells were transfected with scramble siRNA or 30 and 60 pM of NEDD4 siRNA. NEDD4 level was estimated by Western blot, and GAPDH was used as a loading control. (**c**) Bar diagram showing the relative band intensity of the NEDD4 protein. (**d**) After 24 hpt of NEDD4 siRNA, cells were infected with CHIKV (MOI=0.1) followed by collection of cells and supernatants at 12 hpi. Western blot depicting the protein levels of NEDD4 and nsP3. (**e**) The bar diagrams indicating the relative band intensities of nsP3 using Western blot analysis and (**f**) the virus titre from the cell culture supernatants by plaque assay. (**g**) NEDD4 was overexpressed by transfecting NEDD4 plasmid in HEK293T cells, Western blot depicting the level of NEDD4 after overexpression and (**h**) bar diagram showing the relative band intensity of the NEDD4 protein compared to control. (**i**) NEDD4-overexpressed cells were infected with CHIKV (MOI 1), and the levels of nsP3 and NEDD4 were evaluated by Western blot analysis, with a comparison to cells transfected with an empty vector as a control. (**j**) nsP3 protein levels were quantitated by densitometric analysis using the ImageJ software, normalized to GAPDH and plotted as a bar diagram. (**k**) Plaque assay was performed using the infected and infected+NEDD4-overexpressed cell culture supernatants, and the bar diagram depicts the plaque-forming unit in the CHIKV-infected cell supernatant and the NEDD4-overexpressed CHIKV-infected cell supernatant only. The experiments were performed independently in triplicate. The data represent the mean±sd of three independent experiments. *, *P*<0.05; **, *P*<0.01; ***, *P*<0.001; ****, *P*<0.0001; ns, not significant.

To assess the level of NEDD4 during CHIKV infection, Vero cells were infected with CHIKV at a multiplicity of infection (MOI) of 2, and cells were harvested at different time points followed by Western blot. The data demonstrated that NEDD4 level was downregulated in CHIKV-infected cells compared to the mock cells (Fig. 1a), with GAPDH used as a control for expression. This time-course experiment was also conducted in HEK293T cells which showed a non-significant increase in NEDD4 expression at early time points; however, a significant reduction at the later time point was observed after CHIKV infection compared to the uninfected cells (Fig. S1, available in the online Supplementary Material). 

### Modulation of NEDD4 levels affects CHIKV replication

In order to understand the role of NEDD4 in CHIKV infection, siRNA concentrations of 30 and 60 pM were used to silence the expression of the NEDD4 gene in HEK293T cells. After 24 h post-transfection (hpt), cells were harvested and subjected to Western blot analysis (Fig. 1b). The results showed a decrease of 36% and 85%, respectively, in the levels of NEDD4 compared to the scramble siRNA control (Fig. 1c). Subsequently, the siRNA (60 pM)-transfected cells were infected with CHIKV at an MOI of 1.0. The supernatant and the cells were collected at 12 h post-infection (hpi) and processed for plaque assay and Western blot analysis, respectively. The data showed a depletion in the levels of both NEDD4 and CHIKV-nsP3 after siRNA transfection, compared to the scramble control (Fig. 1d). The nsP3 protein level was found to be reduced by 77% after siRNA transfection (Fig. 1e). Similarly, plaque assay revealed a 60% reduction in the viral titre compared to the scrambled siRNA control (Fig. 1f). Collectively, the data suggest that NEDD4 might be one of the important host factors for CHIKV infection.

Next, the effect of NEDD4 overexpression on CHIKV infection was assessed by transfecting 1 µg of NEDD4 plasmid in HEK-293T cells according to the manufacturer’s protocol (Lipofectamine 2000 Reagent Invitrogen). The cells were harvested at 24 hpt and processed for Western blot to measure the level of NEDD4. It was observed that the NEDD4 level was increased by 62% compared to the control (Fig. 1g, h). Next, the NEDD4-overexpressing cells were infected with CHIKV at an MOI of 1.0. The collected supernatant and the cells (after 10 hpi) were subjected to plaque assay and Western blot analysis. An increased level of NEDD4 (96%) was noticed after Western blot analysis in the case of CHIKV infection (Fig. 1i). Interestingly, the level of CHIKV-nsP3 was decreased in the NEDD4-overexpressing cells by 94% (Fig. 1j), where no change of nsP3 was observed in the empty vector transfection control. Further, plaque assay also revealed a 93% reduction in the viral load following NEDD4 overexpression (Fig. 1k). Altogether, these results suggest that the optimum level of NEDD4 is crucial to facilitate efficient CHIKV infection.

### CHIKV-nsP3 interacts with NEDD4 during infection

**Fig. 2.**
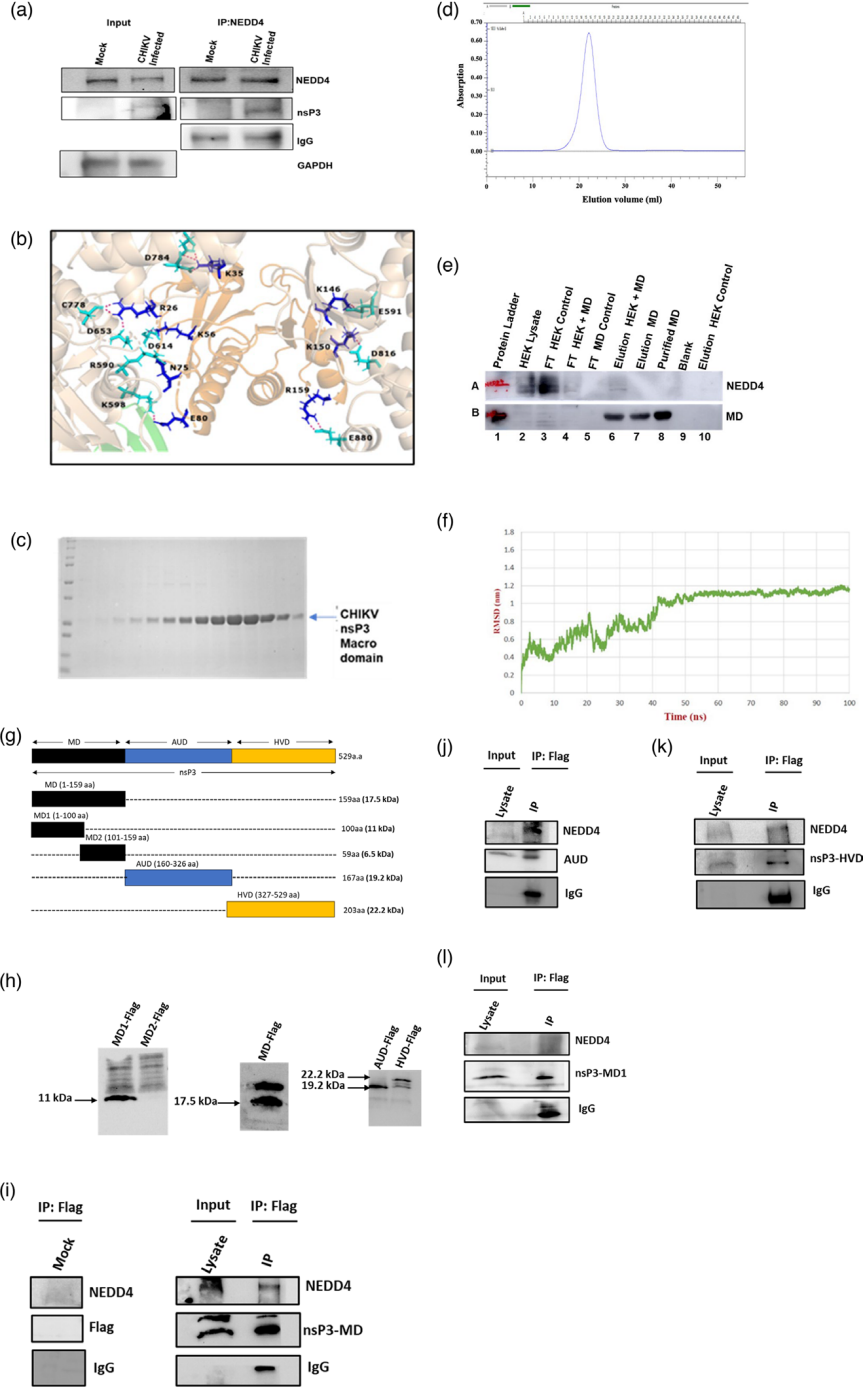
Interaction of host ubiquitin protein NEDD4 with CHIKV-nsP3 protein: (**a**) Western blot analysis of CHIKV-infected Vero cell lysates harvested at 6 hpi following CO-IP with a NEDD4 antibody. The blot shows the levels of host ubiquitin protein NEDD4, nsP3 and GAPDH in both the input and immunoprecipitated samples. (**b**) *In silico* interaction of nsP3-MD with the available crystal structure of NEDD4 (after simulation). (**c**) Coomassie-stained SDS-PAGE showing the purified nsP3-MD using gel filtration chromatography. (**d**) Chromatogram showing the purified nsP3-MD by gel filtration column chromatography. (**e**) Cell lysate of HEK293T cell was incubated with Ni-bead-bound CHIKV-nsP3. The Western blot image showing the presence of NEDD4 and nsP3-MD in different samples collected during the pull-down assay. Lane 1: protein ladder; lane 2: HEK lysate; lane 3: flow-through of HEK lysate binding with Ni-beads; lane 4: flow-through of HEK lysate binding with Ni-bead-bound MD; lane 5: flow-through of purified MD; lane 6: elution of MD-bound HEK lysate; lane 7: elution of Ni-bead-bound purified MD; lane 8: purified MD; lane 9: blank; lane 10: elution of Ni-bead-bound HEK lysate. (**f**) RMSD plot of CHIKV-nsP3 with WW domain of NEDD4 complex at 100 ns timeframe of simulation. (**g**) Schematic representation of various fragments of nsP3 cloned into the pCDNA3.1 vector. (**h**) Western blot showing expression of FLAG-tagged CHIKV nsP3 domains (MD, MD1, MD2, AUD and HVD) in HEK293T cells, by using an anti-FLAG antibody. (h–l) Western blot showing CO-IP of NEDD4 with different FLAG-tagged domains of CHIKV nsP3 using anti-FLAG antibody. HEK293T cells were transfected with individual constructs of nsP3-MD (**h**), AUD (**i**), HVD (**j**) and MD1 (residues 1–100; **k**). Mock-transfected cells served as control. NEDD4 was detected in IP samples from MD, AUD and HVD, but not MD1. All the Western blot experiments were performed independently in triplicate.

Since alterations in the NEDD4 level resulted in a reduction of CHIKV-nsP3 protein level and inhibition of CHIKV infection, its association with nsP3 was investigated. CHIKV-infected Vero cells were harvested at 6 hpi and processed for co-immunoprecipitation (CO-IP), followed by Western blot. It was found that NEDD4 was immunoprecipitated using NEDD4 antibody, and the CHIKV-nsP3 protein was also co-immunoprecipitated with NEDD4 (Fig. 2a), while IgG was used as a negative control. Thus, these results suggest that CHIKV-nsP3 interacts with NEDD4 during CHIKV infection.

To support the above experimental findings, protein–protein docking was carried out using the modelled 3D structure of CHIKV-nsP3 protein [11] with the available crystal structure of the C-terminal part of the human host protein NEDD4 (PDB ID: 5C7J) through the ClusPro server. In order to validate the binding models, all docked complexes were subjected to molecular dynamics simulation analysis over a period of 100 ns, using the WebGro simulation [21]. This interaction study unveiled a robust binding between the human host protein NEDD4 and CHIKV-nsP3. While the majority of interacting residues of the viral protein were from the HVD of nsP3, one, namely, Arg 103, belonged to nsP3-MD. So far, there has not been any report where MD was found to interact directly with any of the host proteins. Therefore, it was interesting to learn more about this involvement of nsP3-MD in interaction with NEDD4. Subsequently, docking experiments were performed with the nsP3-MD, followed by simulation at a 100 ns time scale. The result showed differences between the CHIKV-nsP3-MD-NEDD4 complex and CHIKV-nsP3-NEDD4 complex in terms of interacting amino acids. Post-simulation, only two amino acids in full-length CHIKV-nsP3 were found to be involved in the interaction, notably one, ASP16, within nsP3-MD (Fig. S2a, available in the online Supplementary Material). In contrast, the nsP3-MD-NEDD4 complex displayed a higher number of interacting amino acids (8 aa) after simulation (Fig. 2b and Table S1, available in the online Supplementary Material). This indicates that the C-terminal part of NEDD4 can interact with both nsp3-MD and nsP3. Furthermore, the 100 ns simulation demonstrated a stable complex formation in the case of nsP3-MD-NEDD4, but a weak complex formation between nsP3 and NEDD4 (Fig S2b & c, available in the online Supplementary Material).

### NEDD4 directly binds to CHIK-nsP3MD

To validate the *in silico* findings, nsP3-MD was cloned and expressed in a bacterial system, followed by purification using affinity chromatography and size-exclusion chromatography, respectively. The chromatogram followed by an SDS-PAGE image showed the purified protein concentration (Fig. 2c, d). Further, *in vitro* interaction analysis was performed using the nsP3-MD purified protein and the HEK-293T cell lysate (Fig. 2e). For this, the HEK cell lysate, which contains various cellular proteins, was incubated with nickel (Ni) beads that were pre-bound with the nsP3-MD protein. This setup was designed to test whether any protein in the cell lysate specifically interacted with nsP3-MD. As a control, a parallel incubation was performed where the HEK cell lysate was incubated with unbound Ni beads (Ni beads without nsP3-MD). This control helps to determine if any non-specific binding occurs to the Ni beads themselves. After incubation, the beads were thoroughly washed to remove non-specifically bound proteins. Then, an elution step was performed to release the proteins that were specifically bound to the beads. In the case of the Ni-beads bound with nsP3-MD, the elution step showed the presence of NEDD4 protein (as observed in lane 6, panel A, Fig. 2e) alongside the nsP3-MD protein itself (lane 6, panel B, Fig. 2e). This indicates that NEDD4 was specifically interacting with the nsP3-MD protein. For the control experiment with unbound Ni beads, NEDD4 was not present in the elution but was found in the flow-through (lanes 3 and 10, panel A, Fig. 2e). The flow-through contains proteins that did not bind to the Ni beads and were washed away during the washing steps. This result confirms that NEDD4 interacts with nsP3-MD during CHIKV infection. The faint band of NEDD4 observed in the pull-down assay with nsP3-MD suggests that additional regions of nsP3 may contribute to this interaction. However, experimental validation using purified full-length nsP3 was not feasible due to its instability.

### WW domain of NEDD4 interacts with CHIKV-nsP3

NEDD4 is known to contain WW domains in the middle of the protein, and this is known as the primary site through which it interacts with other proteins [22,23]. These domains mainly interact with proline-rich domains and/or phosphorylated serine and threonine-containing regions of interacting proteins. The AUD and C-terminal domains of nsP3 are proline-rich and also contain several phosphorylated serine and threonine sites [24]. Therefore, it was appropriate to study the interaction between the WW domain of NEDD4 and nsP3. For the *in silico* protein–protein study, the AlphaFold-predicted ribbon model of the WW domain region of NEDD4 (AF-P46934-F1-v4) [25] and the predicted structure of the phosphomimetic mutant of nsP3 [11] were used, followed by molecular dynamics simulation. The simulation plot (Fig. 2f) confirmed a stable interaction between the WW domain and the full-length nsP3 phosphomimetic mutant. It was observed that the residues from all three domains of nsP3-MD, -AUD and -HVD were involved in the interactions with the WW domain, even after the simulation (Fig. S3 and Table S2). In nsP3-MD, the interacting residues are ASP133 and ARG158, which belong to the C-terminal region of nsP3-MD. Altogether, these findings indicated that, in addition to the MD of CHIKV nsP3, AUD and HVD also play a specific role in mediating interactions with the host protein NEDD4.

### NEDD4 interacts with MD, AUD, and HVD domains of CHIKV nsP3

To validate the *in silico* findings, various fragments of nsP3, including the full MD (1–159 aa), a truncated version of MD (MD1, 1–100 aa; MD2, 101–159 aa), AUD (160–326 aa) and HVD (327–529 aa), were cloned into the pCDNA3.1 vector with a FLAG tag (Fig. 2g). These constructs were transfected into HEK293T cells, and expression was confirmed in Western blot analysis using FLAG antibody (Fig. 2h). The MD2 fragment did not express any protein; hence, it was excluded from further analysis. Following this, CO-IP was performed using anti-FLAG antibodies to pull down the nsP3 truncations. The resulting lysates were probed with both anti-NEDD4 and anti-FLAG antibodies. IgG was used as a positive control. NEDD4 was successfully detected in CO-IP experiments with MD (Fig. 2i), AUD (Fig. 2j) and HVD (Fig. 2k), indicating its interaction with all the domains of nsP3. However, NEDD4 was not pulled down with MD1 (Fig. 2l). This supported the *in silico* predictions that the MD1 fragment does not interact with NEDD4, whereas other domains of nsP3 can bind to NEDD4.

## Discussion

In this study, it was observed that an optimum level of NEDD4 is necessary for effective CHIKV infection. In the context of Japanese encephalitis virus (JEV), down-regulation of NEDD4 led to a notable inhibition of infection [26]. Further functional analysis revealed that JEV utilizes NEDD4 to enhance its replication by suppressing viral-induced autophagy. Conversely, overexpression of NEDD4 has been shown to facilitate the release of HIV-1 [27] and promote budding of Ebola virus [28].

Additionally, it was demonstrated that NEDD4 plays a crucial role in facilitating JEV infection specifically in the neuronal cells, and the interaction between the virus and NEDD4 could potentially influence the severity and progress of the disease [26]. However, it has no discernible effect on JEV infection in the non-neuronal cell lines like HUVECs and Huh7. In the current study, it was demonstrated that NEDD4 level is reduced with the progress of CHIKV infection, and this host factor can interact with nsP3 during CHIKV infection. However, its involvement in the disease progression needs to be understood.

Earlier reports suggest that the interaction of nsP3 with host proteins modulates CHIKV infection. For example, it has been shown that Hsp90 interacts with the nsP3 protein in order to enhance viral infection [29]. Furthermore, CHIKV-nsP3 was reported to interact with RM62F, which is involved in a number of gene regulation events, including alternative splicing and RNA release. In addition to this, the interaction of nsP3 and Ago2-RISC complex was also found before [30]. Nevertheless, no evidence of direct interaction of nsP3-MD with any host proteins has been reported to date. In the current study, involvement of nsP3-MD has been observed with NEDD4. Furthermore, both *in silico* and *in vitro* studies suggest that, in addition to the MD domain, the AUD and HVD domains of nsP3 are also required for a stable interaction with NEDD4. In this study, we have only mapped nsP3 for the interaction with NEDD4, but the interacting domains of NEDD4 have not been experimentally confirmed and are only predicted through *in silico* analysis.

The functional significance of these interactions in CHIKV infection needs to be further evaluated. As previously described [18], other viral proteins (nsP1 and nsP4) were also found to interact with NEDD4 following co-transfection. Therefore, investigating the interaction between these viral proteins (nsP1 and nsP4) and NEDD4 will be very informative for a deeper understanding of its role in CHIKV infection.

In conclusion, this study identifies NEDD4 as a key player in CHIKV infection and offers valuable insights into the exploitation of host proteins by viruses. The role of NEDD4 in CHIKV infection, along with its interaction with the residues of nsP3, might be useful to design future therapeutics against CHIKV.

## Supplementary material

10.1099/jgv.0.002136 Uncited Supplementary Material 1.

## Abbreviations

AUD: alphavirus unique domain
CHIKV: Chikungunya virus
CO-IP: co-immunoprecipitation
hpi: hours post-infection
hpt: hours post-transfection
HVD: hypervariable domain
JEV: Japanese encephalitis virus
MD: macrodomain
MOI: multiplicity of infection
Ni: nickel
nsP3-MD: macrodomain of nsP3
