# Supplementary material for: Optimum level of NEDD4 and its interaction with nsP3 are crucial to facilitate efficient Chikungunya virus (CHIKV) infection
Source: J Gen Virol. 2025 Aug 11;106(8):002136. doi: 10.1099/jgv.0.002136 (PMC12451614; doi:10.1099/jgv.0.002136)
Supplement: Uncited Supplementary Material 1. [file jgv-106-02136-s001.pdf]

Supplementary Figures:

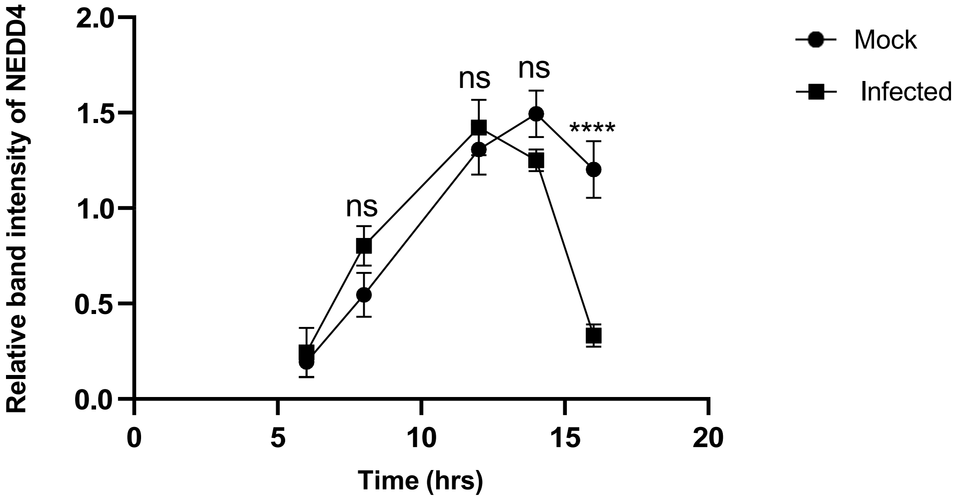

Fig. S1: HEK293T cells were infected with CHIKV and NEDD4 expression was checked at different time points. Line diagram depicting the relative band intensity of NEDD4 in mock and infected cells at different time points.

(a)

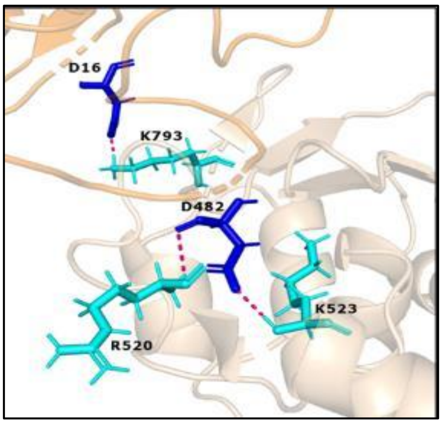

(b)

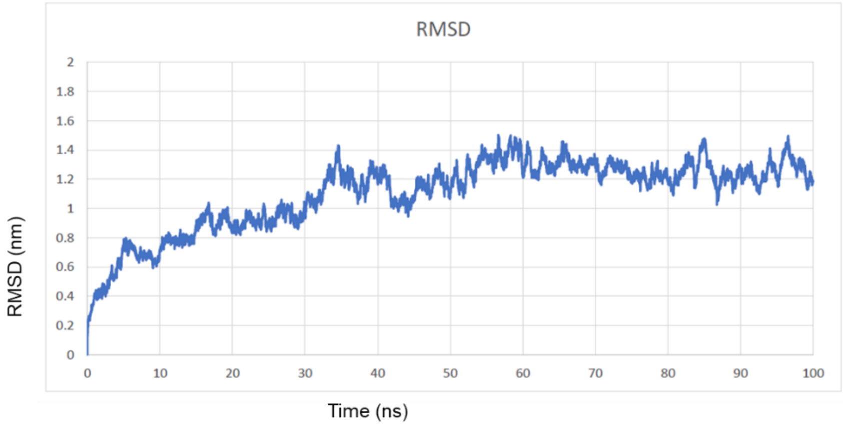

(c)

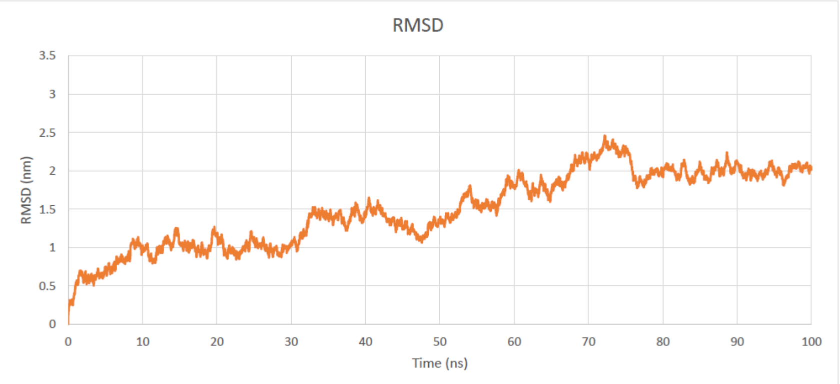

Fig. S2: **(a)** Interaction of ChikV nsP3 with host protein NEDD4 after 100 ns Molecular Docking simulation. **(b)** RMSD plot of CHIKV-nsP3-NEDD4 complex at 100ns; **(c)** RMSD plot of CHIKV-nsP3-MD-NEDD4 complex at 100ns.

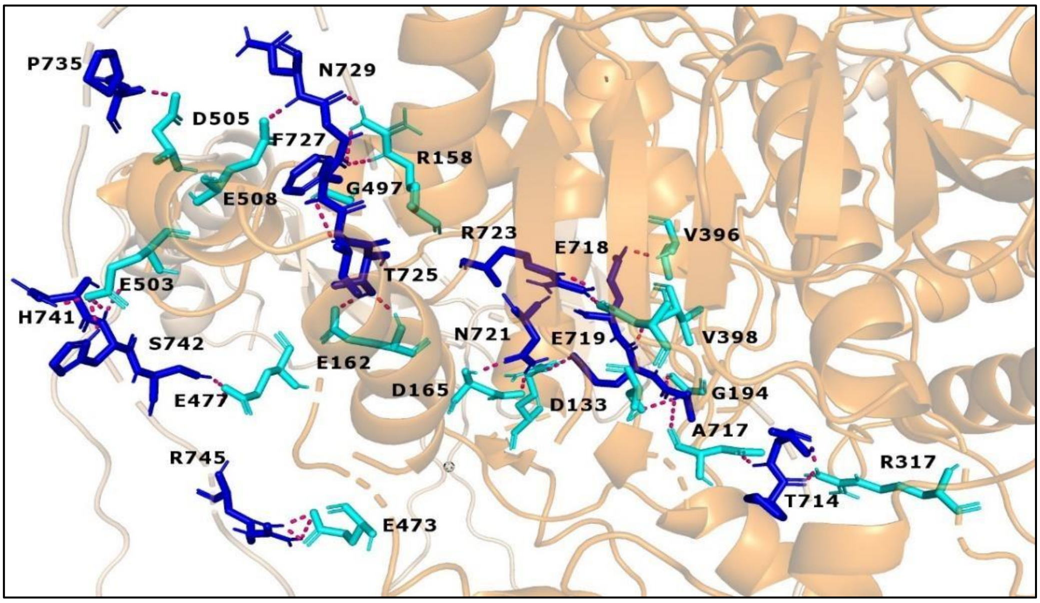

Fig. S3 *In silico* interaction of CHIKV-nsP3 with WW domain of NEDD4 (after simulation). Colour scheme employed: orange represents CHIKV-nsp3, wheat denotes NEDD4. Interacting residues of CHIKV are depicted in blue-coloured sticks, while interacting residues of NEDD4 are represented in cyan-coloured sticks. Hydrogen bonds between the ligand and the protein are illustrated using magenta-coloured dotted lines.

Supplementary Tables:

Serial no.	CHIKV-nsP3-MD	NEDD4
1	GLU80	LYS 598
2	ASN75	ARG590
3	LYS56	ASP614
4	ARG26	ASP 653, CYS778
5	LYS 35	ASP 784
6	LYS 146	GLU 591
7	LYS 150	ASP 816
8	ARG159	GLU 880

Table S1: Amino acid residues involved in the interaction between CHIKV- nsP3-MD and the crystal structure of NEDD4 following simulation analysis.

Serial no.	Interacting residues of nsP3	Domain of ChikV-nsP3 involved in interaction	Interacting residues of WW domain of NEDD4
1	ASP133	MD (MD2)	ARG723
2	ARG158		PHE 727
3	GLU162	AUD	THR725
4	ASP165		ASN721
5	GLY194		ALA717
6	ARG317		THR714
7	HIS376	HVD	SER708
8	VAL396		GLU718
9	VAL398		GLU719
10	GLU473		ARG745
11	GLU477		SER742
12	GLY497		PHE727
13	GLU503		HIS741
14	ASP505		PRO735
15	GLU508		ASN729

Table S2: Amino acid residues involved in the interaction between CHIKV nsP3 and the WW domain of NEDD4 following simulation analysis.
